# Associations Between the Presence of Primary Headaches and Quality of Life in University Students

**DOI:** 10.3390/medicina62030601

**Published:** 2026-03-22

**Authors:** Lukrecija Jakuš, Marina Horvat Tišlar, Ivan Jurak, Mirjana Telebuh, Gordana Grozdek Čovčić, Sonja Jandroković, Darija Mahović

**Affiliations:** 1 Department of Physiotherapy, University of Applied Health Sciences, 10 000 Zagreb, Croatia; lukrecija.jakus@zvu.hr (L.J.); ivan.jurak@zvu.hr (I.J.); mirjana.telebuh@zvu.hr (M.T.); gordana.grozdek-covcic@zvu.hr (G.G.Č.); 2 University Hospital Centre Zagreb, 10 000 Zagreb, Croatia; sonja.jandrokovic@mef.hr (S.J.); darija.mahovic@mef.hr (D.M.); 3 School of Medicine, University of Zagreb, 10 000 Zagreb, Croatia

**Keywords:** primary headaches, university students, quality of life

## Abstract

*Background and Objectives*: Headaches have become one of the global public health burdens in the 21st century. Although findings on the presence of headaches in general and older adult populations have been well-documented, little evidence has been observed for university students. Moreover, their level of quality of life seems to be impaired due to stressful events and the inability to cope with them. However, the mutual relations between headaches and quality of life in this population remain unknown. Therefore, the main purpose of the study was to examine the associations between lifetime headaches and the presence of headaches in the last 12 months with quality of life. *Materials and Methods*: In total, 1350 university students (age = 22.9 ± 2.3 years; 81.3% female) were recruited. Each participant was instructed to fulfill the Headache-Attributed Restriction, Disability, Social Handicap, and Impaired Participation (HARDSHIP) questionnaire, a reliable and valid tool to assess headache and quality-of-life characteristics. Headache characteristics, headache-related disability (HALT-90), and quality-of-life domains were analyzed using Spearman’s correlation analyses and structural equation modeling. *Results*: Participants with migraine reported more frequent and more intense headaches and substantially greater headache-related disability compared with those with tension-type or undifferentiated headache. The mean number of lost days in the previous 90 days (HALT-90) was 14.3 (SD 23.1) in the migraine group compared with 4.53 (SD 12.0) in the tension-type headache group and 5.77 (SD 10.9) in the undifferentiated headache group. Across most WHOQOL domains, students with migraine reported lower quality-of-life scores compared with other headache groups. The WHOQOL-8 total score averaged 30.9 (SD 4.79) in the migraine group and 33.6 (SD 3.93) among participants without headache. Greater headache burden was consistently associated with poorer quality of life, with headache-related disability showing the strongest correlation with energy for everyday life (r = −0.345, *p* < 0.001). *Conclusions*: These findings suggest that greater headache burden, particularly migraine and headache-related disability, is associated with poorer quality of life among university students. The results highlight the need for targeted prevention programs aimed at helping students manage stress more effectively and improve their quality of life.

## 1. Introduction

Headache disorders related to pain, discomfort, or aching have become a global public health burden in the last three decades [1]. Data from the Global Disease Burden Study 2019 indicated that headaches remained the second cause of disability worldwide, especially in women under 50 years of age [2]. The most recent evidence showed that the prevalence of active headache disorders was 52.0%, of which 14.0% and 26.0% were contributed by migraine and tension-type headache (TTH) [3]. Having any type of headache has been associated with behavioral and environmental burdens, including a reduction in productivity and an increase in psychiatric comorbidities, such as stress, anxiety, and depression [4].

Despite the fact that headaches still remain a common non-communicable disorder presented in young and old populations across a lifespan [5,6], the literature highlights that individuals between the ages of 25 and 55 are primarily affected by headaches [7]. Although the general population may be vulnerable to headaches because of their working schedules accompanied by physical and psychological stressors, previous studies have indicated that university students are at increased risk of having headaches [8,9,10,11,12,13]. Estimates suggest that the prevalence of headaches in university students ranges between 9.0% and 28.0%, making them a target group for increased awareness and effective management of headaches [13]. Potential mechanisms related to headaches in university students include accumulated stress and fatigue, fasting, sleep disorders, and an unhealthy lifestyle [14].

The presence of headaches is often associated with a lower level of quality of life [15,16]. The majority of studies conducted among general and older adult populations have shown that individuals who have experienced headaches in their lifetime are at increased risk of a reduced quality of life [15]. Irrespective of the type (migraine or TTH), the recurring pain caused by headaches may influence someone’s physical, psychological, social, and environmental functioning [17]. Despite the effort to examine the associations between headaches and quality of life, most studies have been conducted among the general population [18,19,20], while fewer findings have been evident in university students [13,21,22,23]. Findings have shown that headaches can reinforce overall body pain, sleep deprivation, stress, hunger/skipping meals, and fatigue [23]. Moreover, the same study found that headache sufferers reported moderate to severe disability in daily functioning, limiting their activities as a result of headache attacks [23]. In a study by Rafi et al. (2022) [21], components of quality of life, which included physical inactivity, more screen time, poor sleep quality, and scoring higher on anxiety and depression scales, were considered the most potent factors associated with headaches. In general, it has been confirmed that headaches impact quality of life in university students, impairing their academic performance, decreasing daily activities, missing university school days, and affecting learning and socialization [5,24].

Although these findings suggest that headaches may impact quality of life in university students [13,21,22,23], no study has focused on examining these associations in Croatian university students. A recent study conducted among a large sample of university students showed that a 1-year prevalence of migraines and TTH was between 35.0% and 39.0%, and 91.0% of participants responded positively to the question regarding the incidence of headache in the past 12 months [25]. Given the fact that the prevalence of headaches was higher than previously reported [13], and that Croatian university students had, in general, a lower psychological component of quality of life associated with pain and discomfort [26], it is reasonable to assume that headaches may co-exist with lower levels of quality of life in this population. Also, a high proportion of university students from Croatia is undertaking health-related courses, and they can serve as an avenue to promote and spread the importance of headaches and their impact on quality of life [26]. By examining the relationship between the presence of headaches and quality of life, interventions aiming to target factors for preventing the occurrence of headaches and to increase the level of quality of life are warranted.

Therefore, the main purpose of the study was to investigate the associations between headaches and quality of life in a large sample of health-based Croatian university students. Based on previous findings [5,21], we hypothesized that the presence of headaches would be associated with lower quality of life.

## 2. Materials and Methods

### 2.1. Study Participants and Testing Protocol

In this observational, cross-sectional study, we included 1350 university students enrolled in health profession programs (age = 22.9 ± 2.3 years; 81.3% female), conducted between January and February 2020. Of the 1364 students enrolled in the programs, 1350 completed the questionnaire and were included in the analysis. The participants consisted of university students studying different health profession programs in the 2019/20 academic year at the University of Applied Health Sciences in Zagreb, Croatia. Each participant was requested to fill out the Headache-Attributed Restriction, Disability, Social Handicap, and Impaired Participation (HARDSHIP) questionnaire [27] anonymously and voluntarily. Prior to completing the questionnaire, the participants were informed about the study’s purpose and objectives and provided with detailed instructions for filling out the questionnaire. Individuals who did not attend or were reluctant to take part in the study were omitted. Two participants were removed because of inconsistencies in their responses. The survey was given to participants in small groups of 15–20 students within a university classroom environment. Data collection was conducted by the research team and not by course instructors to minimize potential pressure on students. Once the researcher thoroughly explained the questionnaire and allowed for questions or clarification, every student filled out the questionnaire on their own. The questionnaire took around 15 min to finish. This study was approved by the Ethics Committee of the University of Applied Health Sciences in Zagreb (Class: 602-04/15-18/321; Reg. no.: 251-379-1-15-02).

### 2.2. The HARDSHIP Questionnaire

To estimate the presence of headaches, we used the HARDSHIP questionnaire [27]. The questionnaire was first developed by the Lifting the Burden non-governmental organization to tackle headache screening, sociodemographic information, and factors associated with headache-related burden (symptoms, duration, intensity, and impact on daily living). The data collection was supposed to be face-to-face between a trained interviewer and a participant. The HARDSHIP questionnaire was translated from English to Croatian and back-translated into English for reliability purposes, with sensitivity and specificity properties of >75% for migraine and TTH diagnosis and good to very good agreement (κ = 0.74 to 0.85) [28]. For the purpose of this study, we used questions related to lifetime headaches and headaches in the last 12 months.

Quality-of-life questions were part of the HARDSHIP questionnaire and included items related to quality-of-life satisfaction, daily energy requirements, financial resources for everyday needs, self-rated health, the ability to perform activities on a daily basis, self-satisfaction, personal relationships, and living conditions [27]. Answers to each item were arranged along a 5-point Likert scale from ‘very unsatisfied’ (1) to ‘very satisfied’ (5). Global quality of life was calculated by summing all the scores ranging from 8 to 40, where higher scores denoted better quality of life.

### 2.3. Data Analysis

Descriptive statistics were used to summarize the sample characteristics. Categorical and ordinal variables are presented as frequencies and percentages, while continuous variables are presented as mean and standard deviation (SD), together with median and range (minimum–maximum).

Associations between sex and headache-related variables (lifetime headache, headache in the previous 12 months, headache type, headache frequency, and headache intensity) were examined using Pearson’s chi-square test. Effect sizes were quantified using Cramér’s V.

Spearman’s rank correlation coefficients were calculated to examine associations between headache burden indicators and quality-of-life measures. Headache burden indicators included headache type, HALT-90 lost days, headache frequency, and headache intensity. Correlations were computed for each WHOQOL domain and for the WHOQOL-8 total score.

To account for the ordinal nature of the WHOQOL items and reduce multiple domain-specific regression models, a structural equation model (SEM) was estimated in which the eight WHOQOL items were specified as indicators of a latent quality-of-life factor. Headache type and sex were included as predictors of the latent quality-of-life variable. The SEM approach was chosen to model the measurement structure of the WHOQOL items and to account for measurement error while estimating associations with headache characteristics. Because WHOQOL items are ordinal, the model was estimated using diagonally weighted least squares (DWLS). Model fit was evaluated using the comparative fit index (CFI), Tucker–Lewis index (TLI), root mean square error of approximation (RMSEA), and standardized root mean square residual (SRMR).

All analyses were performed in R, version 4.5.2 [29]. Structural equation modeling was conducted using the “*lavaan*” package [30]. Statistical significance was set at *p* < 0.05.

### 2.4. Results

Sample characteristics by headache type are presented in Table 1. The study included 523 participants with migraine, 483 with tension-type headache, 223 with undifferentiated headache, and 121 without headache. Females were dominant in all headache groups, especially among participants with migraine (87.0%). Only the no-headache group had a more balanced sex distribution (60.3% female).

Participants with migraine reported more frequent and more intense headaches compared with those with tension-type or undifferentiated headache. Over half of migraine sufferers reported headaches several times per month (54.7%), and 25.2% described their headaches as very intense, compared with 4.6% in the tension-type group and 2.2% in the undifferentiated group. Migraine was associated with the greatest disability: the mean number of lost days in the previous 90 days (HALT-90) was 14.3 (SD 23.1) for migraine compared with 4.53 (SD 12.0) for tension-type headache and 5.77 (SD 10.9) for undifferentiated headache.

Across most WHOQOL domains, participants with migraine reported slightly lower mean scores compared with other headache groups, whereas the highest scores were generally observed among participants without headache. The WHOQOL-8 total score averaged 30.9 (SD 4.79) in the migraine group, 32.4 (SD 4.72) in the tension-type group, 31.6 (SD 4.82) in the undifferentiated group, and 33.6 (SD 3.93) among participants without headache.

Sex differences in headache prevalence and characteristics are presented in Table 2. Lifetime headache prevalence was high in both sexes but slightly higher among females (97.0%) than among males (94.5%) (χ^2^ = 3.90, *p* = 0.048), although the effect size was very small (Cramér’s V = 0.054). Similar differences were found in the previous 12 months, with 93.3% of females and 81.0% of males reporting headaches during this period (χ^2^ = 38.23, *p* < 0.001, Cramér’s V = 0.168).

The distribution of headache types also differed significantly by sex (χ^2^ = 46.78, *p* < 0.001, Cramér’s V = 0.186). Migraine was more common among females (41.5%) than among males (26.9%), while males more frequently reported having no headache (19.0% vs. 6.7%).

Spearman correlations between headache burden indicators and WHOQOL variables are presented in Table 3. Overall, a greater headache burden was consistently associated with lower quality-of-life scores. The strongest correlations were observed for HALT-90 lost days, particularly with WHOQOL-2 (energy for everyday life; r = −0.345, *p* < 0.001), WHOQOL-4 (satisfaction with health; r = −0.300, *p* < 0.001), and WHOQOL-5 (satisfaction with ability to perform everyday activities; r = −0.294, *p* < 0.001). Headache frequency and intensity also showed negative correlations with most WHOQOL domains, although the associations were weaker. Headache type showed smaller correlations with quality-of-life domains and was not significantly associated with WHOQOL-6 (satisfaction with oneself) or WHOQOL-7 (satisfaction with personal relationships).

For the overall WHOQOL-8 total score, correlations were negative for all headache burden indicators, with the strongest association observed for HALT-90 (r = −0.324, *p* < 0.001), followed by headache frequency (r = −0.168, *p* < 0.001), headache intensity (r = −0.164, *p* < 0.001), and headache type (r = −0.148, *p* < 0.001). Overall, the observed correlations were small in magnitude.

To account for the ordinal nature of the WHOQOL items and reduce multiple domain-specific analyses, a structural equation model was estimated in which the eight WHOQOL items were specified as indicators of a latent quality-of-life factor. The model demonstrated acceptable overall fit to the data (CFI = 0.985, TLI = 0.991, RMSEA = 0.062, SRMR = 0.051). Standardized factor loadings for the WHOQOL items ranged from 0.53 to 0.81, indicating that all eight items contributed meaningfully to the latent quality-of-life construct (Supplementary Table S1).

Regression coefficients from the structural equation model are presented in Table 4. Compared with the migraine group, participants with tension-type headache (B = 0.238, SE = 0.049, β = 0.155, *p* < 0.001) and those without headache (B = 0.415, SE = 0.081, β = 0.160, *p* < 0.001) had significantly higher latent quality-of-life scores. The difference between the undifferentiated headache and the migraine was not statistically significant (B = 0.104, SE = 0.063, β = 0.052, *p* = 0.097). Male sex was associated with slightly higher quality-of-life scores compared with female sex (B = 0.129, SE = 0.055, β = 0.068, *p* = 0.018). Headache type and sex together explained approximately 4.2% of the variance in latent quality of life.

## 3. Discussion

The main purpose of the study was to investigate the associations between headaches and quality of life in a large sample of health-based Croatian university students. Although the differences in quality-of-life scores between headache groups were generally modest, a consistent pattern of lower perceived well-being was observed among students reporting greater headache burden.

The results revealed a clear overall pattern across headache groups: students with migraine had the greatest headache burden and the lowest quality-of-life scores, whereas participants without headache reported the highest levels of quality of life. Students with tension-type headache generally showed intermediate outcomes, while those with undifferentiated headaches tended to be closer to the migraine group than to the no-headache group. Overall, these findings indicate that greater headache burden, including headache frequency, intensity, and headache-related disability, was associated with poorer quality of life across several WHOQOL domains, particularly those related to self-rated health and energy for everyday life.

This is one of the first studies examining the associations between headaches and quality of life in university students. Our results are in line with previous studies conducted among a similar population of university students [13,21,22,23]. In general, the presence of headaches and greater headache burden have been associated with various physical, psychological, social, and environmental components that may influence quality of life. For example, a study by Rustom et al. (2022) [23] showed that the most triggering predictors of migraines included sleep deprivation, psychological distress, fatigue, and lack of energy to perform well on an everyday basis. The same study revealed that the most common physiological problems resulting from migraine were phonophobia, photophobia, nausea, and vomiting [23]. Interestingly, a systematic review by Axiotidou et al. (2025) [13] pointed out that a lower level of quality of life from frequent headaches was accompanied by poor lifestyle behaviors, including prolonged use of computers and mobile devices, more time spent in sedentary behaviors, physical inactivity, and regular consumption of fast food and drinking coffee.

Migraine clearly stood out as the most burdensome headache type in this population. Compared with other headache groups, students with migraine reported more frequent headaches, greater headache intensity, and substantially higher levels of headache-related disability measured by HALT-90. Migraine was associated with the highest number of lost days in the previous 90 days and the largest proportion of moderate and severe disability grades. These findings indicate that migraine in this student population was not only more symptomatic but also more disruptive to everyday functioning and academic activities.

Quality of life followed a similar gradient across headache groups. Across most WHOQOL domains, students with migraine reported slightly lower mean scores compared with those with tension-type headache, undifferentiated headache, or no headache. The overall WHOQOL-8 total score reflected the same pattern, with the lowest scores observed in the migraine group, somewhat higher scores in the undifferentiated headache group, higher scores in the tension-type headache group, and the highest scores among students without headache. These results suggest that a more severe headache status was associated with poorer perceived quality of life.

Similar observations have been shown in the general population, where individuals without headaches score better in quality of life compared to those with episodic headaches [20]. Lipton et al. (2000) [18] found that the presence of headaches was independently associated with lower physical and mental component scores derived from the SF-36 quality-of-life questionnaire. Our findings extend these observations by showing that, within a student population, migraine is associated with a greater headache burden and poorer quality of life compared with other headache types. Interestingly, we found that the strongest predictors of headaches were associated with self-rated health and the lack of energy important for daily activities, which could be attributed to both physical and psychological components of quality of life. On the other hand, previous evidence has suggested that the presence of headaches is more often associated with psychological quality of life [18]. This would indicate that individuals with worsening headache problems and potential disabilities may be more prone to reductions in mental health and increases in depression and anxiety states. In Canadian men and women, findings showed that those with mild, moderate, or severe disability levels of headache scored notably lower in physical and psychological quality-of-life component scores compared to controls without the headaches [16].

The associations between headache burden indicators and quality of life further supported this pattern. In the correlation analyses, greater headache burden was consistently related to lower WHOQOL scores. Among the burden indicators examined, headache-related disability measured by HALT-90 showed the strongest correlations with quality-of-life domains, particularly with energy for everyday life, satisfaction with health, and the ability to perform daily activities. Although headache frequency and intensity were also negatively associated with quality of life, their associations were weaker, while headache type itself showed smaller correlations with WHOQOL domains. This suggests that the functional impact of headaches, such as lost days and limitations in daily functioning, may be especially important for understanding reduced quality of life among students.

Sex differences were also observed in the distribution of headaches. Headaches were common in both sexes but were reported more frequently among female students, particularly in the previous 12 months. Migraines were also more prevalent among women, while male students more often reported having no headache. Women were more likely to report frequent and intense headaches, whereas men more often reported headaches occurring only occasionally. Although these differences were statistically significant, the effect sizes were relatively small, suggesting that the pattern is consistent but not large in magnitude.

Another important aspect relates to the magnitude of the observed associations. Although several correlations between headache burden indicators and quality-of-life domains reached statistical significance, the effect sizes were generally small. Given the relatively large sample size of the present study, even modest associations may achieve statistical significance. Therefore, the findings should be interpreted with caution, as the clinical significance of these effects may be limited.

The multivariable structural equation model confirmed the same general pattern observed in the descriptive and correlational analyses. When the eight WHOQOL items were modeled as a latent quality-of-life construct, students with tension-type headache and those without headache had significantly higher quality-of-life scores compared with students with migraine. In contrast, the difference between migraine and undifferentiated headache was not statistically significant, suggesting that undifferentiated headache may resemble migraine more closely in terms of its association with quality of life. Male students also reported slightly higher quality-of-life scores than female students. However, headache type and sex together explained only a small proportion of the variance in quality of life, suggesting that additional behavioral, psychological, and environmental factors may also contribute to students’ well-being. Although the structural equation model identified statistically significant associations between headache type and quality of life, the proportion of explained variance was relatively small (4.2%). This indicates that headache type and sex account for only a limited part of the variability in quality of life among university students. Therefore, while headaches appear to be associated with poorer quality of life, they should not be interpreted as the primary determinant of well-being in this population. Rather, headaches are likely to represent only one component within a broader constellation of behavioral, psychological, and environmental factors influencing students’ quality of life.

Headaches still remain a global public health burden related to different biological, behavioral, and environmental factors. High prevalence of self-reported headaches in student populations has also been reported in previous epidemiological studies, which may partly reflect the inclusion of occasional or mild headache episodes captured by questionnaire-based assessments [31]. A population of university students may be at extreme risk of headaches because of their inability to cope with everyday distress and tasks [25]. The transition period between adolescence and adulthood during university years is often accompanied by making personal decisions, studying, or reading, which may impact academic performance, decrease daily physical activities, and increase time spent in sedentary behaviors [13]. Since the prevalence of headaches is constantly rising and can be up to 20% [13,25], and the level of quality of life in university students may be poorer than in other populations [26], the co-existence between headaches and quality of life in this specific population has yet to be investigated. Overall, our findings suggest that headaches, particularly migraines and headaches associated with greater disability, may represent an important marker of reduced well-being among university students. The results highlight that the functional impact of headaches, reflected in disability and lost days, may be especially relevant when interpreting how headaches influence quality of life in young adults. It should also be acknowledged that migraine and tension-type headache represent primary headache disorders with established neurobiological and pathophysiological mechanisms. Consequently, while behavioral and psychosocial factors typical of student populations may contribute to headache burden, the occurrence of these conditions cannot be fully attributed to lifestyle-related determinants alone. The modest proportion of explained variance observed in the present model likely reflects the multifactorial determinants of quality of life rather than the absence of clinically meaningful effects of primary headache disorders.

Despite the effort to examine the associations between headaches and quality of life, the findings should be interpreted considering several limitations. First, we collected the data from a single sample at the University of Applied Health Sciences, while university students from other health-related courses were not considered. As such, the results of this study may not be applicable to university students from other specialties, and the generalizability of the findings should be interpreted with caution. Second, we used a self-reported questionnaire to collect data regarding socio-demographic characteristics and those related to headaches and quality of life using the HARDSHIP questionnaire [27]. Although this questionnaire has been validated in a Croatian sample of university students [28], we relied on participants’ self-reporting of headaches, which were not verified clinically. Finally, we used a cross-sectional design to examine the associations between headaches and quality of life, which does not allow for conclusions about causality. Future research should therefore include longitudinal designs and clinical verification of headache diagnoses to better understand the temporal relationship between headaches and quality of life in student populations.

In addition, the analyses were adjusted only for sex. Although the HARDSHIP questionnaire collects detailed information on headache characteristics and headache-related burden—including headache type, frequency, intensity, duration, and headache-attributed disability measured using HALT-90 as well as selected quality-of-life indicators—it does not include a comprehensive set of behavioral, psychological, or socioeconomic variables. Consequently, potentially relevant factors such as academic workload, examination periods, sleep patterns, psychological stress, symptoms of anxiety or depression, physical activity, caffeine consumption, screen exposure, and socioeconomic conditions were not available for inclusion in the analytical models. Because these variables were not collected within the framework of the original questionnaire, their potential confounding effects could not be examined. Therefore, the observed associations between headache burden and quality of life should be interpreted with caution.

The present study was conducted on a large sample of university students representing a relatively homogeneous age group in early adulthood. Although the observed differences in quality of life associated with headache burden were generally modest, the findings indicate that even within this relatively healthy and functionally active population, headaches are associated with measurable deviations in perceived well-being. It is possible that larger differences in quality of life may emerge later in life as individuals experience changes in social and occupational roles, increasing work-related responsibilities, and cumulative health burdens. Future research should therefore examine these relationships across different stages of adulthood and consider both cross-sectional and longitudinal study designs to better understand how the impact of headache disorders on quality of life evolves over time. Taken together, these findings suggest that headache burden may represent an early indicator of reduced well-being even in relatively young and otherwise healthy populations, highlighting the importance of early recognition and prevention strategies.

## 4. Conclusions

In summary, we found that lifetime headaches and headaches reported in the last 12 months were significantly and negatively associated with quality-of-life domains and general quality of life in a large sample of health-related university students. Those with headaches had poorer quality of life, especially in terms of self-rated health and the energy for everyday tasks. This study adds to the existing body of evidence that headaches are associated with lower levels of quality of life in university students. Based on these findings, policymakers should create prevention programs to help university students at increased risk of headaches cope with stressful events more efficiently in order to increase their quality of life. Although the observed effect sizes were generally modest, the findings from this large student sample indicate that headache burden—particularly migraine and headache-related disability measured by HALT-90—is consistently associated with poorer perceived well-being. These results suggest that headache burden may represent an early indicator of reduced quality of life even in relatively young populations. Future research using longitudinal designs and including a broader range of behavioral and psychosocial variables may provide further insight into how the impact of headache disorders on quality of life evolves across different stages of adulthood.

## Figures and Tables

**Table 1:** Sample characteristics, HALT-90, and WHOQoL-8 domain scores by headache type.

	Migraine (N = 523)	Tension-Type (N = 483)	Undifferentiated (N = 223)	No Headache (N = 121)
Sex				
Female	455 (87.0%)	386 (79.9%)	183 (82.1%)	73 (60.3%)
Male	68 (13.0%)	97 (20.1%)	40 (17.9%)	48 (39.7%)
Headache (lifetime)				
Yes	523 (100%)	483 (100%)	223 (100%)	74 (61.2%)
No	0 (0%)	0 (0%)	0 (0%)	47 (38.8%)
Headache (12 months)				
Yes	523 (100%)	483 (100%)	223 (100%)	0 (0%)
No	0 (0%)	0 (0%)	0 (0%)	121 (100%)
Headache frequency				
Several times a year	222 (42.4%)	316 (65.4%)	97 (43.5%)	-
Several times a month	286 (54.7%)	139 (28.8%)	51 (22.9%)	-
Every day	4 (0.8%)	1 (0.2%)	2 (0.9%)	-
Missing	11 (2.1%)	27 (5.6%)	73 (32.7%)	-
Headache intensity				
Not intense	24 (4.6%)	124 (25.7%)	27 (12.1%)	-
Moderate	367 (70.2%)	335 (69.4%)	113 (50.7%)	-
Very intense	132 (25.2%)	22 (4.6%)	5 (2.2%)	-
Missing	0 (0%)	2 (0.4%)	78 (35.0%)	-
Headache frequency				
HALT-90 lost days				
Mean (SD)	14.3 (23.1)	4.53 (12.0)	5.77 (10.9)	-
Median [Min, Max]	7.00 [0, 240]	0 [0, 160]	1.50 [0, 60.0]	-
Missing	83 (15.9%)	81 (16.8%)	37 (16.6%)	-
HALT-90 disability grade				
Minimal or infrequent	203 (38.8%)	311 (64.4%)	131 (58.7%)	-
Mild or infrequent	66 (12.6%)	43 (8.9%)	26 (11.7%)	-
Moderate	88 (16.8%)	33 (6.8%)	18 (8.1%)	-
Severe	83 (15.9%)	15 (3.1%)	11 (4.9%)	-
Missing	83 (15.9%)	81 (16.8%)	37 (16.6%)	-
WHOQoL-1				
Mean (SD)	4.03 (0.734)	4.18 (0.766)	4.06 (0.800)	4.34 (0.665)
Median [Min, Max]	4.00 [1.00, 5.00]	4.00 [1.00, 5.00]	4.00 [1.00, 5.00]	4.00 [2.00, 5.00]
WHOQoL-2				
Mean (SD)	3.52 (0.885)	3.80 (0.866)	3.67 (0.883)	4.07 (0.755)
Median [Min, Max]	4.00 [1.00, 5.00]	4.00 [1.00, 5.00]	4.00 [1.00, 5.00]	4.00 [2.00, 5.00]
WHOQoL-3				
Mean (SD)	3.72 (0.999)	3.95 (0.960)	3.83 (0.946)	4.04 (0.879)
Median [Min, Max]	4.00 [1.00, 5.00]	4.00 [1.00, 5.00]	4.00 [1.00, 5.00]	4.00 [1.00, 5.00]
WHOQoL-4				
Mean (SD)	3.71 (0.871)	3.99 (0.865)	3.89 (0.837)	4.30 (0.703)
Median [Min, Max]	4.00 [1.00, 5.00]	4.00 [1.00, 5.00]	4.00 [1.00, 5.00]	4.00 [2.00, 5.00]
WHOQoL-5				
Mean (SD)	3.89 (0.936)	4.07 (0.852)	3.94 (0.881)	4.24 (0.817)
Median [Min, Max]	4.00 [1.00, 5.00]	4.00 [1.00, 5.00]	4.00 [1.00, 5.00]	4.00 [1.00, 5.00]
WHOQoL-6				
Mean (SD)	3.81 (0.898)	3.99 (0.763)	3.84 (0.853)	4.03 (0.694)
Median [Min, Max]	4.00 [1.00, 5.00]	4.00 [1.00, 5.00]	4.00 [1.00, 5.00]	4.00 [2.00, 5.00]
WHOQoL-7				
Mean (SD)	3.98 (0.800)	4.07 (0.778)	4.01 (0.814)	4.07 (0.755)
Median [Min, Max]	4.00 [1.00, 5.00]	4.00 [1.00, 5.00]	4.00 [1.00, 5.00]	4.00 [2.00, 5.00]
WHOQoL-8				
Mean (SD)	4.24 (0.847)	4.36 (0.816)	4.37 (0.754)	4.46 (0.606)
Median [Min, Max]	4.00 [1.00, 5.00]	5.00 [1.00, 5.00]	4.00 [1.00, 5.00]	5.00 [3.00, 5.00]
WHOQoL-8 total score				
Mean (SD)	30.9 (4.79)	32.4 (4.72)	31.6 (4.82)	33.6 (3.93)
Median [Min, Max]	31.0 [15.0, 40.0]	33.0 [8.00, 40.0]	32.0 [11.0, 40.0]	34.0 [23.0, 40.0]

Legend: WHOQoL-1 = quality of life; WHOQoL-2 = energy for everyday life; WHOQoL-3 = money for needs; WHOQoL-4 = satisfaction with health; WHOQoL-5 = satisfaction with ability to perform everyday activities; WHOQoL-6 = satisfaction with oneself; WHOQoL-7 = satisfaction with personal relationships; WHOQoL-8 = satisfaction with living conditions; WHOQoL-8 total score = total score represents the sum of all eight domains.

**Table 2:** Headache prevalence, headache type, headache frequency, and headache intensity distribution by sex.

Variable	Female *n* (%)	Male *n* (%)	χ^2^	*p*-Value	Cramér’s V
Lifetime headache					
Yes	1064 (97.0%)	239 (94.5%)	3.90	0.048	0.054
No	33 (3.0%)	14 (5.5%)			
Headache in the last 12 months					
Yes	1024 (93.3%)	205 (81.0%)	38.23	<0.001	0.168
No	73 (6.7%	48 (19.0%)			
Headache type					
Migraine	455 (41.5%)	68 (26.9%)	46.78	<0.001	0.186
Tension-type	386 (35.2%)	97 (38.3%)			
Undifferentiated	183 (16.7%)	40 (15.8%)			
No headache:	73 (6.7%)	48 (19.0%)			

**Table 3:** Spearman correlations between headache burden indicators and WHOQOL variables.

WHOQOL	Headache Type	HALT-90	Frequency	Intensity
WHOQOL-1	−0.096 (<0.001)	−0.222 (<0.001)	−0.121 (<0.001)	−0.135 (<0.001)
WHOQOL-2	−0.165 (<0.001)	−0.345 (<0.001)	−0.176 (<0.001)	−0.199 (<0.001)
WHOQOL-3	−0.091 (<0.001)	−0.190 (<0.001)	−0.093 (0.002)	−0.093 (0.002)
WHOQOL-4	−0.186 (<0.001)	−0.300 (<0.001)	−0.179 (<0.001)	−0.160 (<0.001)
WHOQOL-5	−0.087 (0.001)	−0.294 (<0.001)	−0.132 (<0.001)	−0.128 (<0.001)
WHOQOL-6	−0.052 (0.057)	−0.189 (<0.001)	−0.075 (0.012)	−0.053 (0.073)
WHOQOL-7	−0.037 (0.176)	−0.159 (<0.001)	−0.100 (<0.001)	−0.068 (0.022)
WHOQOL-8	−0.084 (0.002)	−0.163 (<0.001)	−0.073 (0.015)	−0.104 (<0.001)
WHOQOL total	−0.148 (<0.001)	−0.324 (<0.001)	−0.168 (<0.001)	−0.164 (<0.001)

Legend: All values are r (*p*).

**Table 4:** Structural equation model of latent quality of life predicted by headache type and sex.

Predictor	B	SE	β (Standardized)	*p*-Value
Tension-type headache (vs. migraine)	0.238	0.049	0.155	<0.001
Undifferentiated headache (vs. migraine)	0.104	0.063	0.052	0.097
No headache (vs. migraine)	0.415	0.081	0.160	<0.001
Male (vs. female)	0.129	0.055	0.068	0.018

Note: Latent variable = overall quality of life defined by the eight WHOQOL items.

## Data Availability

The data presented in this study are available on reasonable request from the corresponding author. The data are not publicly available due to ethical and privacy restrictions in accordance with GDPR regulations.

## Supplementary Materials

The following supporting information can be downloaded at https://www.mdpi.com/article/10.3390/medicina62030601/s1, Table S1: Standardized factor loadings for the WHOQOL-8 items.
