# Supplementary material for: Associations Between the Presence of Primary Headaches and Quality of Life in University Students
Source: Medicina (Kaunas). 2026 Mar 22;62(3):601. doi: 10.3390/medicina62030601 (PMC13027642; doi:10.3390/medicina62030601)
Supplement: Supplementary file 1 [file medicina-62-00601-s001.zip › medicina-4187821-supplementary.pdf]

Table S1. Standardized factor loadings for the WHOQOL-8 items

WHOQOL item	Standardized loading
WHOQOL-1	0.731
WHOQOL-2	0.717
WHOQOL-3	0.534
WHOQOL-4	0.727
WHOQOL-5	0.779
WHOQOL-6	0.808
WHOQOL-7	0.752
WHOQOL-8	0.696
